# Editorial: ticks & tick-borne parasites and diseases

**DOI:** 10.1017/S0031182024001549

**Published:** 2024-08

**Authors:** Ala E. Tabor

**Affiliations:** 1 The University of Queensland, Queensland Alliance for Agriculture & Food Innovation, Centre for Animal Science, 80 Carmody Road, St. Lucia 4072, Queensland, Australia; 2 The University of Queensland, School of Chemistry and Molecular Biosciences, 68 Cooper Road, St. Lucia 4072, Queensland, Australia

**Keywords:** ecology, epidemiology, taxonomy, tick parasites, tick-borne diseases, ticks, vaccines

## Abstract

Ticks and tick-borne diseases affect humans, livestock, and wildlife in most regions of the globe. Although there are over 900 tick species globally, only approximately 10% of species are second to mosquitoes as major vectors of human and veterinary diseases. The 17 articles of this themed Special Issue highlight the current research trends associated with newly discovered tick species, concepts of tick evolution, new vaccinology approaches, factors affecting disease transmission, and factors affecting tick ecology and tick-borne disease epidemiology. Table 1 summarizes the articles in this Special Issue in alphabetical author order and Fig. 1 is a word cloud generated from the article titles. Of the 17 articles in this Special Issue, two are review articles (vaccinology) while the remaining 15 are original research articles. The topics range from tick control, to epidemiology, ecology, tick-borne disease control, tick-borne disease transmission, vaccine approaches, and the description of novel extant and extinct tick species. Fig. 2 is graphical representation of the articles within this Special Issue including tick hosts and the most representative tick species studied. The articles also include authors from most continents globally with first author contributions from Australia, Bangladesh, Brazil, Czech Republic, Germany, India, Mexico, Pakistan, South Africa, Spain, Turkey, United Arab Emirates, USA and Zambia. This issue is thus truly diverse which reflects the diversity of ticks, tick-borne diseases and they hosts they infest globally.

## Graphical abstract

**Figure:**
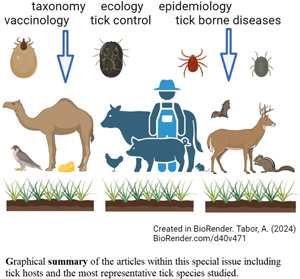

## Tick taxonomy

Ticks are divided into three extant families, Ixodidae or hard ticks 731-742 species (Dantas-Torres 2018; Guglielmone *et al.*, 2020), Argasidae or soft ticks 216 species (Dantas-Torres 2018; Mans *et al.*, 2019), and Nuttalliellidae (single species *Nuttalliella namaqua* limited to the Afrotropic region, Latif *et al.*, 2012). The latter family has aspects of both soft and hard ticks and has been described as the evolutionary link between these two large families. Thus, phylogenetic and taxonomic studies continue to better understand how the current extant tick species evolved. In this Special Issue, Chitimia-Dobler *et al*. (2024b) examine extinct tick species found in amber to suggest that the distribution of *Nuttalliella* likely stretched from Africa over Antarctica and much of Australia before the rift with Burma at ~150 mya (https://doi.org/10.1017/S0031182024000477). From eight fossils in Burmese amber the Nuttalliellidae were found to be comprised of three genera: *Deinocroton*, *Legionaris* nov. gen. and *Nuttalliella*, and the following new species: *Deinocroton bicornis* sp. nov.; *Deinocroton lacrimus* sp. nov.; *Nuttalliella gratae* sp. nov.; *Nuttalliella tuberculata* sp. nov. *Nuttalliella placaventrala* sp. no*.; Nuttalliella odyssea* sp. nov.; *Nuttalliella tropicasylvae* sp. nov.; and *Legionaris robustus* sp. nov. The authors suspect that the Australian continent may have extant Nuttalliellidae yet to be discovered. A separate study in this Special Issue by Mans *et al.* (2024) using mitochondrial genome and nuclear ribosomal RNA sequencing, demonstrated that *Alveonasus* genus is paraphyletic and that *Alveonasus lahorensis* is better placed within the soft tick sub-family of Argasinae rather than Ornithodorinae (https://doi.org/10.1017/S0031182024000441). In addition, after sampling animal shelters in Khyber Pakhtunkhwa in Pakistan, Ali *et al.* (2024) identified a new tick species using mitogenome sequencing and morphological comparisons: *Ornithodoros pakistanensis* sp. nov. in the Pavlovskyella subgenus (https://doi.org/10.1017/S0031182024000982). Applying similar mitogenomic sequencing methods and morphological identifications, Chitimia-Dobler *et al.* (2024a) discovered a new hard tick species from Eleonora's falcons on Antikythira Island in Greece (https://doi.org/10.1017/S0031182024000866): *Ornithophysalis* subgenus *Haemaphysalis doenitzi*. The significance of this finding is that this falcon species is a long-distance migrant of the Afro-Palearctic flyway breeding during summer in the Mediterranean and winter in North-West Africa, and this is the first identification of this tick genus in the Western Palearctic region.

## Tick and TBD vector control

Lyme disease (borreliosis) is the most prevalent vector-borne disease in both Europe and the United States, presenting a significant public health concern. The main causative agent in the United States is *Borrelia burgdorferi* transmitted by *Ixodes scapularis* ticks, while the predominant species in Europe are *Borrelia afzelii*, *Borrelia garinii*, and *B. burgdorferi* transmitted by *Ixodes ricinus* (Marques *et al*., 2021). Ostfeld *et al*. 2024 (https://doi.org/10.1017/S0031182024000349) examined the effects of acaricide treatments in 24 residential neighbourhoods of Dutchess County (New York, USA) on the subsequent pathogen coinfection in *I. scapularis* ticks known to carry multiple medically important pathogens such as *Anaplasma phagocytophilum*, *Babesia microti* and *B. burgdorferi*. The use of fungus based biopesticides showed coinfections of *B. microti* and *B. burgdorferi* to be more common than single infections. However, when using tick control system bait boxes, the bias towards coinfections was eliminated. The authors concluded that control methods directed at ticks attached to small mammals may influence human exposure to coinfected ticks and the probability of exposure to multiple tick-borne infections. Chemical acaricides have proven effective in reducing tick infestation loads on livestock and pets primarily targeting the tick central nervous system (Obaid *et al*., 2022). In previous studies, passive topical application of fipronil significantly reduced the burden of nymphs and larvae of *I. scapularis* on small reservoir hosts and decreased the abundance of nymphs in treated areas. In addition, infection rates of *B. burgdorferi* and *A. phagocytophilum* in reservoir animals were significantly reduced after treatment (Dolan *et al*., 2004, 2016). Šíma *et al*. (2024) used a mouse model to demonstrate the nanomolar efficiency of Fipronil (phenylpyrazole chemical class) against *I. ricinus* ticks and its rapid speed-or-kill aimed at blocking the transmission of *B. afzelii* pathogens (https://doi.org/10.1017/S0031182024001136).

**Table 1.** Summary of the 17 articles included in this Special Issue ‘Ticks & Tick-Borne Parasites and Diseases’ and the section title associated with this Editorial

Authors	Article Title	Section title in Editorial
Ali *et al*.	Description of a new Ornithodoros (Pavlovskyella) (Ixodida: Argasidae) tick species from Pakistan.	Tick Taxonomy
Butler *et al*.	Ecological relationships of *Haemaphysalis longicornis* Neumann with other tick species on wildlife hosts at cowcalf farms implementing integrated pest management in eastern Tennessee.	Epidemiology and Ecology
Chitimia-Dobler *et al*.	Discovery of a novel Mediterranean *Haemaphysalis (Ornithophysalis) doenitzi* group tick species infesting *Falco eleonorae* on Antikythira Island, Greece.	Tick Taxonomy
Chitimia-Dobler *et al*.	Nuttalliellidae in Burmese amber: implications for tick evolution.	Tick Taxonomy
de la Fuente and Ghosh	Evolution of tick vaccinology.	Vaccinology
Ferreira *et al*.	Glycine rich proteins of ticks: more than a cement component	Vaccinology
Godinho *et al*.	Ecology and phenology of the bat tick *Argas* (*Carios*) *dewae* (Acari: Argasidae).	Epidemiology and Ecology
Kuyucu and Hekimoglu	Predicting the distribution of *Ixodes ricinus* in Europe: integrating microclimatic factors into ecological niche models.	Epidemiology and Ecology
Mans *et al*.	Mitochondrial genome and nuclear ribosomal RNA analysis place *Alveonasus lahorensis* within the Argasinae and suggest that the genus *Alveonasus* is paraphyletic.	Tick Taxonomy
Moraes *et al*.	Aurora kinase as a putative target to tick control.	Tick and TBD Vector Control
Munjita *et al*.	*Rhipicephalus simus* ticks: New hosts for phleboviruses.	Tick and TBD Vector Control
Ostfeld *et al*.	Effects of residential acaricide treatments on patterns of pathogen coinfection in blacklegged ticks.	Tick and TBD Vector Control
Perez-Soria *et al*.	Immunization of cattle with a *Rhipicephalus microplus* chitinase peptide containing predicted B-cell epitopes reduces tick biological fitness	Vaccinology
Willingham *et al*.	Camel tick species distribution in Saudi Arabia and United Arab Emirates using MaxEnt modelling.	Epidemiology and Ecology
Shakya *et al*.	Assessment of farmers' knowledge, attitudes and control practices (KAP) to mitigate acaricide resistance and tick borne diseases.	Tick and TBD Vector Control
Šíma *et al*.	Fipronil prevents transmission of Lyme disease spirochetes.	Tick and TBD Vector Control
Zim *et al*.	First seroprevalence survey of bovine anaplasmosis: an emerging tick-borne disease in commercial livestock and dairy farms in Bangladesh.	Epidemiology and Ecology

*Rhipicephalus simus*, classified within the genus *Rhipicephalus* and the family Ixodidae is a highly capable vector of pathogens of critical importance in both medical and veterinary fields (Shekede *et al*., 2021; Phiri *et al*., 2023). This hard tick species not only thrives in diverse habitats but also exhibits a remarkable ability to infest and feed on humans, thus potentially facilitating the transmission of a wide range of infectious agents (Horak *et al*., 2002). Phleboviruses belonging to the genus Phlebovirus and family Phenuiviridae are frequently identified in ticks of the genus *Rhipicephalus* worldwide (Li *et al*., 2016; Pereira *et al*., 2017; López *et al*., 2020) but have not been reported in *R. simus*. Tick-borne phleboviruses (TBPVs) were largely neglected until recently when severe fever with thrombocytopenia syndrome virus (SFTSV) and Heartland virus (HRTV) were confirmed as causative agents of severe disease in humans (McMullan *et al*., 2012; Li *et al*., 2016). Munjita *et al*. 2024 (https://doi.org/10.1017/S0031182024001033) used metagenomic next-generation sequencing to determine the viral diversity in tick populations from a dormant commercial farm in the riverine area in Lusaka, Zambia. This is the first report of a phlebovirus found in *R. simus* ticks.

The knowledge, attitudes and control practices of farmers in the Dhar district of Madhya Pradesh (India) was assessed by Jamra *et al*. (2024) to mitigate acaricide resistance and tick-borne diseases covering 200 livestock owners using a questionnaire (https://doi.org/10.1017/S0031182024001331). Jamra *et al*. (2024) concluded that 75% of respondents were not aware of TBDs and that 36.5% showed favourable attitudes towards adopting tick control practises. In addition, grazing animals were six times more susceptible to ticks compared to livestock held in mixed feeding or manger systems. *Rhipicephalus microplus* and *Hyalomma anatolicum* ticks most commonly affecting livestock in India (Ghosh *et al*., 2006) were assessed and found to be resistant to deltamethrin in all five different sub-divisions due to the easy availability of this acaricide. The study recommended the development of targeted educational programs to enhance farmers' knowledge of sustainable tick control practices to explore alternatives to chemical acaricides to minimise acaricide resistance and TBDs in livestock.

**Figure 1.**
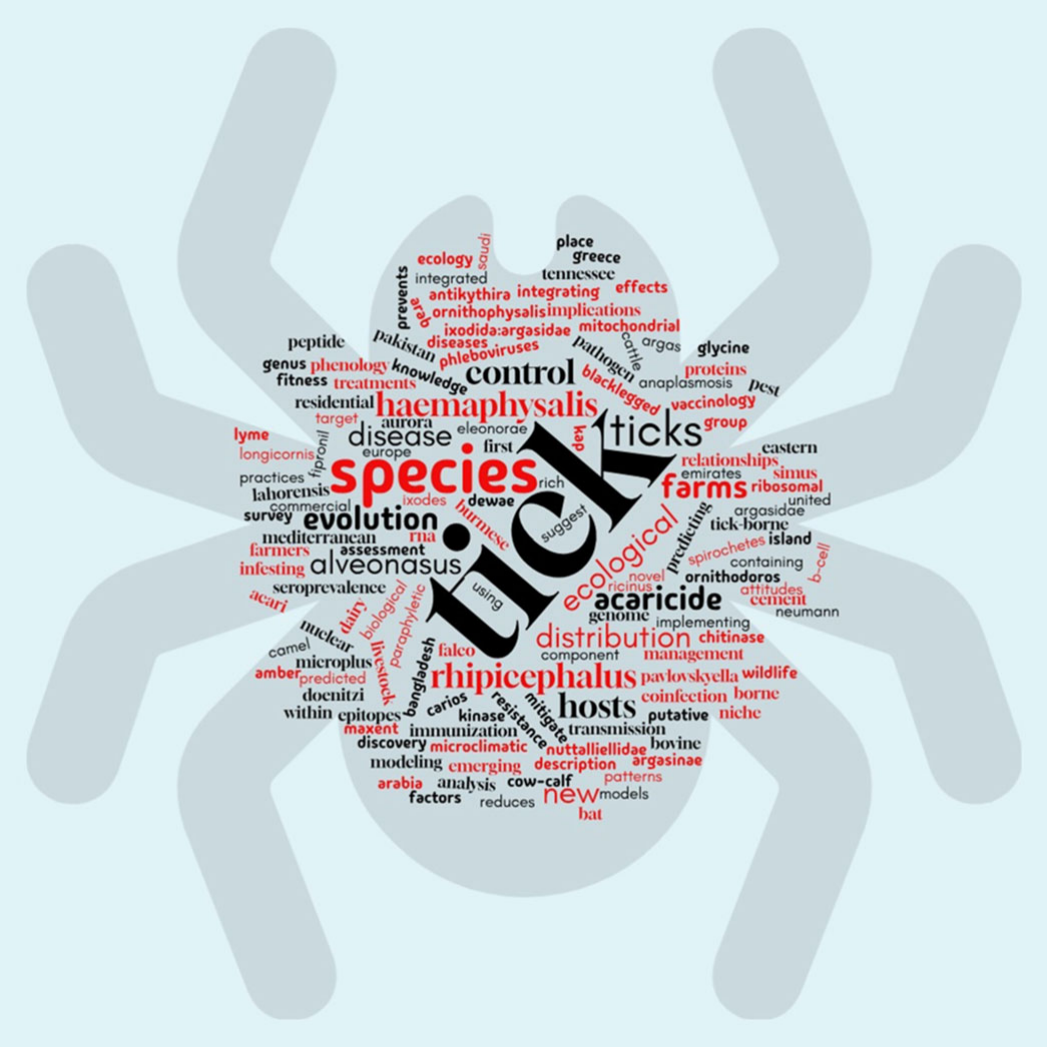
Word cloud generated using the article titles in this Special Issue – Ticks & Tick-borne Parasites and Diseases.

Using the BME26 tick embryonic cell line (Esteves *et al*., 2008), Moraes *et al*. 2024 (https://doi.org/10.1017/S003118202400101X) targeted *R. microplus* aurora kinases (AURK) using a pan AURK inhibitor (CCT137690). AURK play a central role in controlling the cell cycle in a range of organisms and belong to the family of serine-threonine kinase proteins. Their roles in the cell cycle include entry into mitosis, maturation of the centrosome and formation of the mitotic spindle. The authors identified two AURK coding sequences in the transcriptome of *R. microplus* (Rm-AURKA and Rm-AURKB) and cell viability decline was demonstrated in BME26 cells using the pan AURK inhibitor. The authors suggest that AURK inhibitors could be exploited to develop species specific tick control strategies.

## Epidemiology and ecology

Enzyme-linked-immunosorbent-assays (ELISAs) to determine the seroprevalence of bovine tick fever pathogens have been use for almost 30 years in various regions of the world. Countries with high live cattle export industries have routinely vaccinated using the milder *Anaplasma centrale* for bovine anaplasmosis and attenuated strains of *Babesia bigemina* and *Babesia bovis* (reviewed by Salinas-Estrella *et al*., 2022). South Africa, Australia, Argentina, Brazil, Uruguay, and Israel have used *A. centrale* to control *A. marginale* infections. Zim *et al*. 2024 (https://doi.org/10.1017/S0031182024001495) have demonstrated the emergence of bovine anaplasmosis in commercial livestock and dairy farms in Bangladesh and may consider vaccination as a future control measure.

Several studies in this Issue have investigated or predicted the ecological spread of four different tick species in four different geographical regions respectively. The recent U.S. invasion of *Haemaphysalis longicornis* (longhorn tick) has led to studies of human and livestock tick-borne disease transmission and its relationship with wildlife tick species on affected cattle farms. Butler *et al*. 2024 (https://doi.org/10.1017/S0031182024001380) concluded that farmer controlled integrated pest management strategies, and the reduction of tick populations led to better tick management. In Europe, the spread of the castor bean tick *I. ricinus* (significant vector of various diseases including Lyme borreliosis to humans) was determined using microclimatic and macroclimatic models (https://doi.org/10.1017/S003118202400132X). Through the application of this mixed modelling, Kuyucu and Hekimoglu (2024) suggest significant expansion of *I. ricinus* into northern and eastern Europe, with declines in southern Europe. In Saudi Arabia and United Arab Emirates, *Hyalomma dromedarii* is the most abundant tick species affecting primarily camels and other livestock to a lesser extent. Maximum Entropy Species Distribution Modelling (MaxEnt.) used species presence, land use/landcover, elevation, slope and 19 bioclimatic variables to model current and future distribution of *H. dromedarii* ticks (https://doi.org/10.1017/S0031182024001161). Willingham *et al*. (2024) highlighted those areas in the north, east and south-western parts that were highly suitable for this tick species. Finally, Godinho *et al*. 2024 (https://doi.org/10.1017/S0031182024000817) studied the ecology of one of the 12 native soft tick species (Argasidae) in Australia, *Argas dewae*. This tick parasitises several insectivorous bat species and has also been recorded on humans. *A. dewae* populations were monitored on two bat hosts (*Chalinolobus gouldii*; *Austronomus australis*) at three sites in the southern state of Victoria for 28 months showed that tick load increased throughout winter and peaked in the first month of spring before remaining low during late spring and summer. This paper also reports the first records of *A. dewae* from six bat species in three bat families (Miniopteridae; Molossidae; Vespertilionidae) and a second record of *A. dewae* from a human. Godinho *et al*., also document the first distribution records for *A. dewae* in an additional three Australian states. This data will contribute to improvements in wildlife health management and public health preparedness.

**Figure 2.**
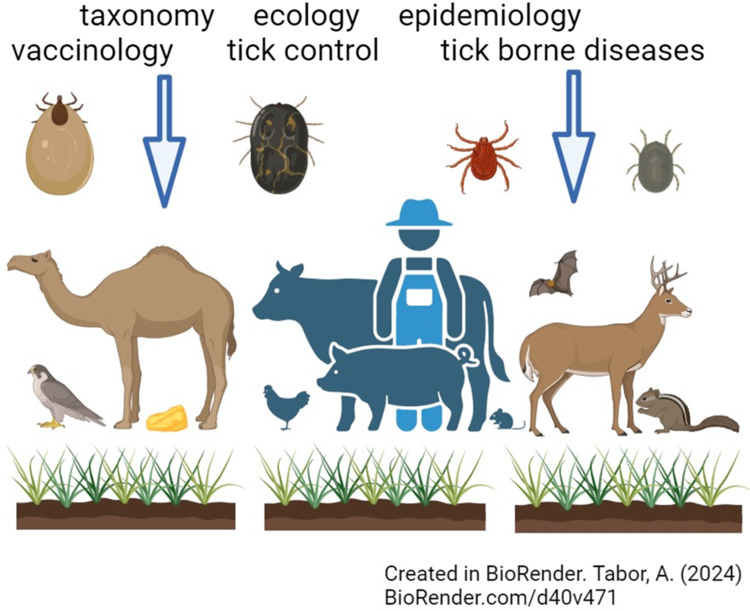
Graphical representation of articles collected for the ‘Ticks & Tick-borne Parasites and Diseases’ Special Issue demonstrating the tick species (Hard tick species Ixodidae: *Ixodes* spp., *Rhipicephalus* spp., *Haemaphysalis* spp., Soft tick species: Argasidae), the hosts and the main topics covered by the article collection.

## Vaccinology

Three articles in this Special Issue reviewed tick vaccinology (de la Fuente and Ghosh, 2024) or described potential vaccine antigens. *Rhipicephalus microplus* is the most significant tick species impacting livestock industries worldwide estimated at USD22-30b annually (Lew-Tabor and Rodriguez-Valle, 2016). Overreliance on chemical treatments for tick control has led to the emergence of acaricide-resistant ticks and environmental contamination while vaccine strategies offer an alternative approach for tick control. Perez-Soria *et al*. 2024 (https://doi.org/10.1017/S0031182024000143) predicted four *R. microplus* B-cell epitopes based on the enzyme chitinase. Chitinases degrade older chitin at the time of tick moulting. Immunization experiments demonstrated that Chitinase peptide 3 reduced weight and oviposition of engorged ticks and reduced larval viability at a 71% overall vaccine efficacy.

Ferreira *et al*. 2024 (https://doi.org/10.1017/S0031182024001410) reviewed tick glycine-rich proteins (GRPs). The authors described the functions of tick GRPs historically associated with salivary gland secretion to form the tick cement cone enabling host attachment and highlighted other GRP roles. GRPs have been identified in a diverse array of organisms and shown to possess several distinctive biological characteristics, including nucleic acid binding, adhesive glue-like properties, antimicrobial activity, involvement in the stress response and in the formation of cuticle components. The authors highlight that GRPs are present in all tick developmental stages, and that expression is modulated by physiological processes and immune challenges such as feeding and pathogen infection. The authors further discuss possible roles of tick GRPs and highlight the vaccine potential of these proteins by summarizing published vaccination experiments in rabbits, mice, cattle and guinea pigs against *H. longicornis*, *Rhipicephalus haemaphysaloides*, *R. microplus* or *Rhipicephalus appendiculatus* ticks.

Finally, de la Fuente and Ghosh (https://doi.org/10.1017/S003118202400043X) describe the challenges of tick vaccines including: (1) Ticks are difficult to control, (2) Vaccines control tick infestations by reducing ectoparasite fitness and reproduction, (3) Vaccine efficacy against multiple tick species, (4) Impact of tick strain genetic diversity on vaccine efficacy, (5) Antigen combination to improve vaccine efficacy, (6) Vaccine formulations and delivery platforms and (7) Combination of vaccines with transgenesis and para-transgenesis. Their review suggests that advances in tick organ antigen recombinant proteins and chimeras designed using vaccinomics and quantum vaccinomics will be combined with technologies such as multi-omics, AI and Big Data, mRNA vaccines, microbiota-driven probiotics and vaccines. In addition, the authors predict that tick vaccines could be combined with other interventions associated with regional ticks' infestations and tick-borne diseases for a personalized medicine approach.

## Data Availability

All data used in the study is disclosed in the paper and corresponding references.
